# In Situ Polymerization of Antibacterial Modification Polyamide 66 with Au@Cu_2_O-ZnO Ternary Heterojunction

**DOI:** 10.3390/polym16010158

**Published:** 2024-01-04

**Authors:** Xiang Li, Mi Zheng, Shikun Zhao, Zhiwen Cao, Kai Pan, Xinxing Feng, Hua Zhang, Min Zheng, Cheng Wang

**Affiliations:** 1 Institute of System Engineering, Academy of Military Sciences, People’s Liberation Army, Beijing 100010, China; 13220298273@163.com (X.L.); feng-xinxing@hotmail.com (X.F.); 2 College of Textile and Clothing Engineering, Soochow University, Suzhou 215123, China; zhengm666@foxmail.com (M.Z.); wangcheng971027@163.com (C.W.); 3 College of Materials Science and Engineering, Beijing University of Chemical Technology, Beijing 100029, China; wsadzsk@live.com (S.Z.); caozhiwen0514@163.com (Z.C.); pankai@mail.buct.edu.cn (K.P.)

**Keywords:** polyamide 66, in situ polymerization, Au@Cu_2_O-ZnO ternary heterojunction, antibacterial

## Abstract

In situ polymerization has proven to be an effective route through which to introduce function materials into polyamide materials. In this work, a nano-heterojunction material was evenly dispersed in PA66 via in situ polymerization methods to yield the antimicrobial PA66. The composites showed excellent antibacterial activity against *Staphylococcus aureus* and *Escherichia coli,* with strong mechanical properties. Fourier transform infrared spectroscopy (FTIR) showed that metal ions reacted with oxygen-containing functional groups. In addition, the shift of oxygen peaks in XPS spectra confirmed the occurrence of a complexation reaction. X-ray diffraction (XRD) and differential scanning calorimetry (DSC) confirmed the effect of nano-heterojunction, which induced crystallization. Scanning electron microscopy (SEM) and transmission electron microscopy (TEM) showed uniform dispersion of heterojunctions in PA66. Tensile testing revealed decreased toughness with higher loadings. The nanocomposite polyamide material has good processing properties which can be processed into thin films, molds, and wires without changing the morphology, and can be widely used in a variety of fields.

## 1. Introduction

Polyamide 6,6 (PA66) stands as a prominent member of the group of polyamide materials, holding a significant presence in the consumer market as one of the largest families of engineering plastics [1,2]. Over the past decades, its remarkable mechanical properties have found extensive applications in engineering plastics, films, and textile fibers, establishing it as a versatile material of choice in various industries [3,4,5]. In recent years, with the development of medical and health undertakings, antibacterial materials have been a topic of research. The dramatic increase in demand for personal protective equipment, including masks and gloves, has coincided with a significant rise in the quest for antimicrobial agents and antimicrobial personal protective equipment [6,7,8]. Thus, the focus on PA66 and other polyamide fabrics has gone beyond mere comfort to include health management functions, with a particular focus on their antimicrobial properties [9].

Photocatalytic antibacterial technology has gained significant attention as a safe and effective antimicrobial technique [10,11,12]. Unlike conventional approaches relying on metal ion leaching for bactericidal action, this process generates active oxygen species (ROS) through photocatalysis on semiconductor surfaces, effectively exterminating bacteria [13]. This technology is expected to be a solution for the antimicrobial modification of polyamide materials.

The integration of heterojunction, combining diverse materials such as metals, conductive carbon materials, and semiconductors, offers promising prospects for the elevation of photocatalytic performance [14]. This stems from their ability to expand the spectral absorption range of the photocatalytic material and enhance charge carrier separation through favorable interface effects. Two notable photocatalysts, wide band gap zinc oxide (ZnO) (band gap of 3.2 eV) and narrow band gap cuprous oxide (Cu_2_O) (band gap of 2.2 eV), have garnered attention for their exceptional photocatalytic efficacy, abundant availability, cost-effectiveness in synthesis, and low toxicity [15,16]. The strategic fabrication of ZnO/Cu_2_O heterojunction has emerged as a viable approach to achieve superior photocatalytic performance. This configuration induces an irreversible electric field at the p-n interface, effectively suppressing the recombination of photoinduced electron–hole pairs.

In addition to semiconductor coupling, the hybridization of noble metal nanoparticles, such as Ag, Au, and Pt, holds great potential for elevating catalytic performance through the formation of Schottky barriers [17,18,19]. This unique phenomenon effectively hinders the recombination of photogenerated electrons and holes, resulting in significantly improved catalytic efficiency [20,21,22]. Among the noble metals, Au emerges as the preferred choice over Ag due to Ag’s susceptibility to oxidation into Ag_2_O under heating or ultraviolet irradiation, causing color changes and diminished antibacterial properties. In contrast, Au nanoparticles exhibit superior chemical stability and demonstrate the most pronounced localized surface plasmon resonance (LSPR) effects, making them highly desirable candidates for catalytic applications [14,23,24,25,26]. Building on this concept, Yuan et al. successfully developed ZnO/Au@Cu_2_O heterojunction using Au nanoparticles supported on ZnO nanorods as seeds for the in situ growth of Cu_2_O shells. Under simulated sunlight exposure for 45 to 90 min, the photocatalytic degradation efficiencies of methyl orange and rhodamine B were remarkably improved, reaching 93.0% and 99.6%, respectively. These results far exceeded the performance of catalysts composed of either a single component or two components [27]. While few reports on the Au@Cu_2_O/ZnO ternary heterojunction nanomaterials for achieving antibacterial properties in PA66.

In this study, we innovatively fabricated an Au@Cu_2_O-ZnO ternary heterojunction, the first of its kind. Following this, we applied an in situ polymerization technique to seamlessly incorporate the Au@Cu_2_O/ZnO heterojunction into the PA66 matrix. This integration resulted in the composite material exhibiting not only exceptional antibacterial properties, but also strong mechanical strength. Furthermore, we conducted an in-depth analysis of the distribution of nano-sized Au@Cu_2_O/ZnO within the PA66 matrix. This analysis revealed significant insights into the changes in the crystalline structure of the resulting PA66 polymers due to the incorporation of the heterojunction. The successful integration of Au@Cu_2_O/ZnO into the PA66 matrix signifies a major advancement with promising applications in diverse fields, notably in the creation of biomedical textiles and protective clothing. This development will potentially set the stage for future innovations in material science and industry-specific applications.

## 2. Experimental Section

### 2.1. Materials

Polyamide 66 salt was supplied by Jianhu County Xinglong PA66 Co., Ltd., Yancheng, China. cupric chloride (CuCl_2_), Sodium dodecyl sulfate (SDS), sodium hydroxide (NaOH), hydroxylamine hydrochloride ethyl alcohol, adipic acid (ADA), 1, 6-Hexamethylenediamine (HMDA), sulfuric acid (H_2_SO_4_), and other chemical reagents used in our experiment were purchased from Shanghai Maclin Biochemical Technology Co., Ltd., Shanghai China. Deionized water was supplied by Jiahua Special PA66 Co., Ltd., Jiaxing China. *Escherichia coli* (*E. coli*, ATCC 25922) and *Staphylococcus aureus* (*S. aureus*, ATCC 6538) were provided by the Guangdong Institute of Microbiology (Guangzhou, China). All of the materials were of commercial grade and were used as received, without further purification.

### 2.2. Preparation of Au@Cu_2_O-ZnO

In the synthesis of the Au@Cu_2_O@ZnO ternary heterojunction, we started by dissolving 0.1 g of CuCl_2_ in 180 mL of ultra-pure water. Then, 2.02 g of sodium dodecyl sulfate (SDS) was added and completely dissolved, followed by the addition of 6 mL of gold nanorods (Au NRs) solution and 6 mL of 1 M NaOH solution. Subsequently, 0.14 g of hydroxylamine hydrochloride in a 10 mL aqueous solution was added under rapid agitation. After allowing the mixture to precipitate for 2 h, it underwent filtration, water washing, and alcohol washing. The Au@Cu_2_O was finally obtained through vacuum drying at 60 °C for 12 h. The ternary heterojunction Au@Cu_2_O@ZnO was then produced using an ultrasonic and microwave co-reactor by adding 0.06 g of the Au@Cu_2_O nanopowder into a 100 mL composite solution containing 0.5 M Zn^2+^, then stirring for 2 h with ultrasonication.

### 2.3. Preparation of PA66/Au@Cu_2_O-ZnO Samples

Samples were meticulously prepared in a controlled environment. The process began in a GSH-5L polymerization autoclave specifically designed for such operations, which was equipped with mechanical agitation to ensure even mixing. To create an inert atmosphere, which is essential for the polymerization process, the entire system was purged with nitrogen gas for 20 min, effectively eliminating any air or potential contaminants. The initial phase of the experiment involved the careful addition of precisely weighed polyamide 66 salt, a novel Au@Cu_2_O-ZnO heterojunction, and high-purity deionized water into the autoclave. This mixture was then stirred at a consistent speed of 60 revolutions per minute, establishing a homogeneous solution for the ensuing polymerization.

To initiate the aqueous solution polymerization process, the autoclave’s internal environment was carefully heated to a temperature of 220 °C. Concurrently, the pressure relief valve of the autoclave was finely adjusted to maintain a constant pressure of 2.0 MPa. This controlled environment was crucial for the reaction to proceed smoothly, allowing for the gradual formation of oligomers over a period of 2 h. In the subsequent melting condensation polymerization stage, the system underwent gradual decompression to reach atmospheric pressure, while the temperature was simultaneously increased to 265 °C. This stage was critical in order for the polymer chains to lengthen and be strengthened. The polymerization process was then continued under a vacuum, at an elevated temperature of 275 °C, for a duration of 40–60 min, ensuring thorough polymerization. Upon completion of the polymerization process, the final product was carefully extracted from the autoclave and processed into chips for further analysis. In this experiment, polyamide 66 (PA66) samples were prepared with varying concentrations of the Au@Cu_2_O-ZnO heterojunction, specifically at mass fractions of 400 ppm, 800 ppm, and 1200 ppm. These samples were designated as PA66/Au@Cu_2_O-ZnO-x, where ‘x’ represents the specific antibacterial content. Additionally, a control sample of pure PA66, devoid of any filler, was also synthesized for comparative purposes.

**Figure 1:**
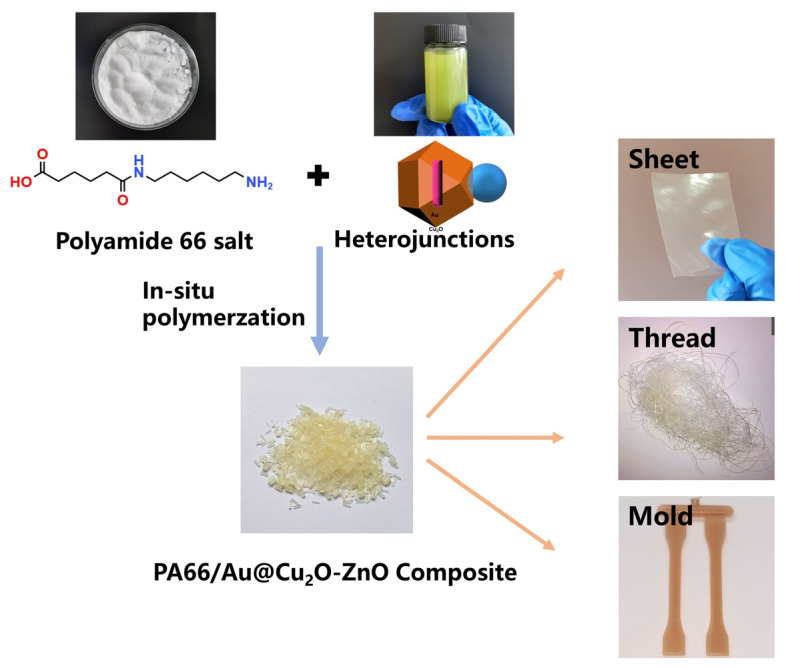
The preparation of PA66/Au@Cu_2_O-ZnO composites.

After the polymerization was completed, the sample was cut into slices using a pelletizer and then micro-injected using a twin-screw injection molding machine. The film samples (~0.20 mm) were prepared by hot pressing for 3 min at a temperature of 275 °C under a holding pressure of ~10 MPa. (Figure 1)

### 2.4. Characterization

Fourier transform infrared analysis (FTIR) was performed using Nicolet 6700 FTIR (Thermo Fisher Scientific, Waltham, MA, USA) with a range of 600–4000 cm^−1^ and a resolution of 4 cm^−1^. X-ray diffraction (XRD) of the pure and PA66/Au@Cu_2_O-ZnO was conducted using an X’Pert PRO Powder Diffractometer (Cu Kα radiation 1.5406 Å, 40 kV, 40 mA) in the range of 5°–80° with a 2θ scale(PANalytical B.V., Almelo, Netherland). An X-ray photoelectron spectroscopy (XPS) test was performed using an Escalab 250Xi system (Thermo Fisher Scientific, Waltham, MA, USA). Relative viscosity measurement followed the ISO 307 standard [28] for testing. The test solution (0.01 g mL^−1^) was prepared by dissolving the sample in concentrated sulfuric acid. After a 12-hour stabilization period, its relative viscosity was measured using an unbeholden viscometer in a constant temperature water bath at 25 °C. The relative viscosity was determined by comparing the flow times of the solvent and the solution. Differential scanning calorimetry (DSC) was performed on a TA Q2000 instrument with 5–10 mg samples(TA Instruments, New Castle, DE, USA). The samples were heated to 300 °C at 30 °C min^−1^ under a 50 mL min^−1^ nitrogen flow and held for 10 min to eliminate thermal history. Then, they were cooled to 100 °C at 10 °C min^−1^ and reheated to 300 °C at the same rate. Thermogravimetric analysis (TGA) was conducted using a TA SDT Q50 instrument(TA Instruments, New Castle, DE, USA) heated to 800 °C at a heating rate of 10 °C min^−1^ under a nitrogen atmosphere. The surface morphology of the composites was characterized by a Carl Zeiss Ultra-55 instrument (Carl Zeiss, Oberkochen, Germany) and transmission electron microscopy (TEM, FEI Tecnai G20, Hillsboro, OR, USA). Tensile testing was carried out at room temperature using an Instron 365 material testing machine (Instron, Boston, MA, USA), according to ISO 527-1:2012 [29].

### 2.5. Antibacterial Activity of PA66/Au@Cu_2_O-ZnO Samples

Methodology for the antibacterial test: Antibacterial activity was assessed against an array of two bacterial strains: *S. aureus* (ATCC 6538) as Gram-positive bacteria and *Escherichia coli* (ATCC 25922) as Gram-negative bacteria. The antibacterial rates were determined according to GB/T 23763-2009 [30]. Each experiment was performed in triplicate, and all values are expressed as means ± standard error.

## 3. Results and Discussions

### 3.1. The Morphology of Au@Cu_2_O-ZnO

**Figure 2:**
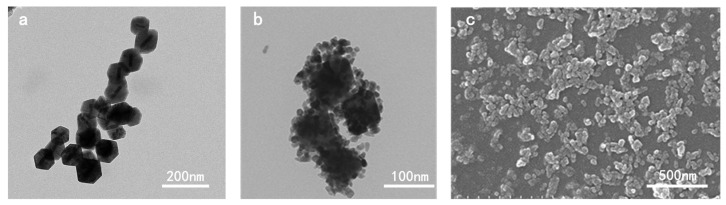
(**a**) TEM image of Au@Cu_2_O heterostructures; (**b**) TEM image of Au@Cu_2_O@ZnO ternary heterojunction; (**c**) SEM image of Au@Cu_2_O@ZnO ternary heterojunction.

Coupling 65 × 20 nm gold nanorods with 0.07% hydroxylamine hydrochloride yielded 97 nm, truncated octahedral Au@Cu_2_O heterostructures with uniform dispersion, and a strong visible light response was attained. Au@Cu_2_O@ZnO ternary heterojunction with even shell coverage was prepared successfully through adding an Au@Cu_2_O heterostructure into a 0.01 M zinc salt reaction system, under certain pH and temperature. The TEM and SEM morphology of Au@Cu_2_O and Au@Cu_2_O@ZnO ternary heterojunction proved the octahedral shape of the Au@Cu_2_O binary heterostructures and Au@Cu_2_O@ZnO ternary heterojunction. In addition, the Au@Cu_2_O was covered successfully by ZnO nanoparticles (Figure 2). Moreover, the scanning spectra in Figure S1 demonstrate that the sample had a shell of a truncated octahedron, indicating an average size of around 110 nm. From Figure S1c, it is clearly visible that the core–shell nanoparticles contained only one gold nanorod, with no excess cores and only the presence of Cu_2_O particles, confirming the successful preparation of these core–shell-structured nanoheterostructures. Figure S1e further confirms the presence of gold rods through SAED analysis. The XRD spectrum in Figure S2 confirms that ZnO was successfully attached to the Au@Cu_2_O surface.

### 3.2. Characterization of PA66/Au@Cu_2_O-ZnO Composites

**Figure 3:**
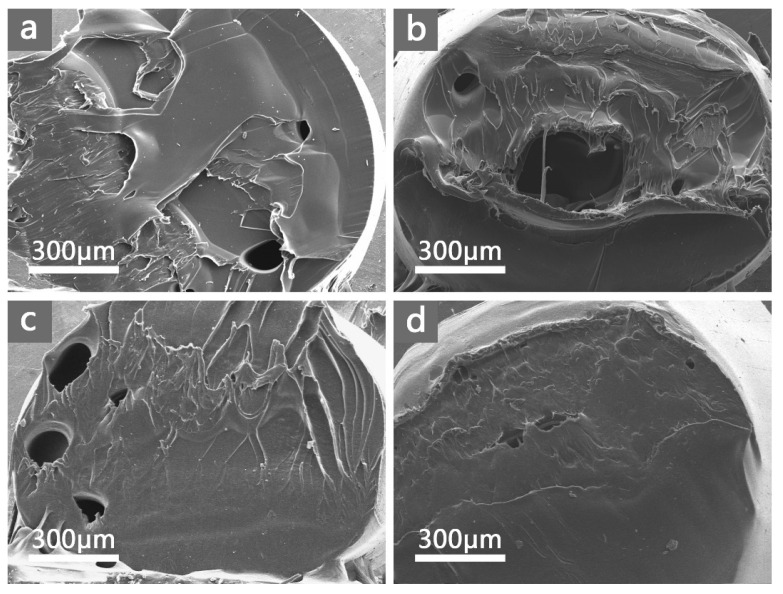
SEM images of fracture sections: (**a**) pure PA66; (**b**) 400 ppm; (**c**) 800 ppm; (**d**) 1200 ppm.

The interfacial compatibility of antibacterial agents in the polymer matrix significantly influences the properties of composite materials. Therefore, scanning electron microscopy (SEM) was used to monitor the cross-section portion of the PA66/Au@Cu_2_O-ZnO composite, and the results are presented in Figure 3a–d. Remarkably, no noticeable differences were observed among the fracture sections of the PA66 or PA66/Au@Cu_2_O-ZnO composites. All samples exhibited a homogenous phase, and no two-phase structure was evident in the PA66/Au@Cu_2_O-ZnO composites, indicating excellent dispersion of PA66/Au@Cu_2_O-ZnO in the PA66 matrix. The strong complexation between metal ions and oxygen-containing functional groups in the heterostructured nanomaterials likely accounts for the robust interfacial compatibility [31].

**Figure 4:**
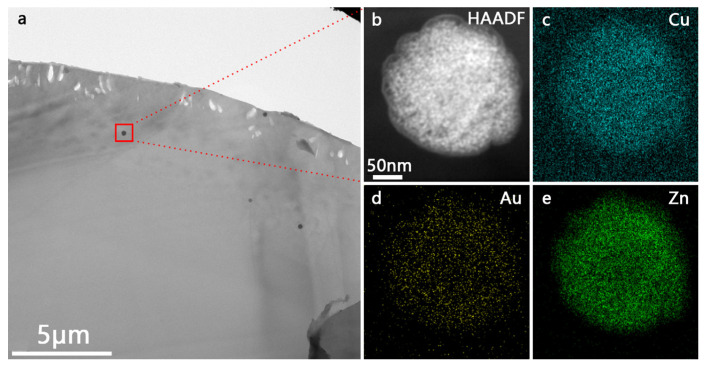
TEM images of fracture sections: (**a**) PA66/Au@Cu_2_O-ZnO; (**b**–**e**) elemental mapping images of (**c**) Cu, (**d**) Au, and (**e**) Zn corresponding to (**b**) Au@Cu_2_O-ZnO compositions.

According to SEM-EDS characterization (Figures S3 and S4 and Table S1), the PA66/Au@Cu_2_O-ZnO composite material was shown to be uniformly dispersed. Furthermore, Figure 4 reveals some black particles on the surface of the material. The elemental analysis of these particles (Figure 4b–e) clearly reveals the presence of Au, Cu, and Zn, indicating some aggregation of the nanoheterostructures. All of these findings confirm that Au@Cu_2_O-ZnO was successfully loaded onto the PA66 substrate.

**Figure 5:**
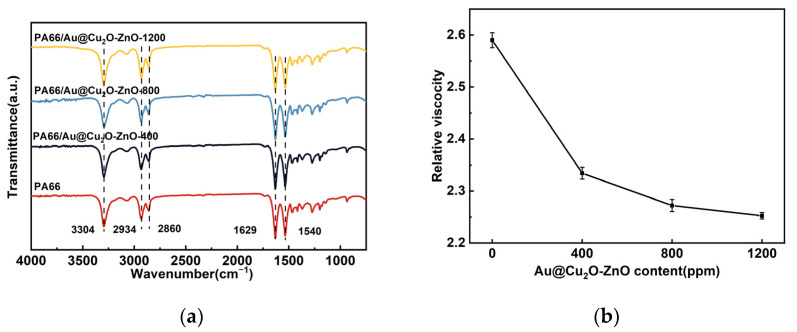
(**a**) FTIR spectrums of PA66 and PA66/Au@Cu_2_O-ZnO composites; (**b**) relative viscosities of PA66 and PA66/Au@Cu_2_O-ZnO composites.

Figure 5a illustrates the FTIR-ATR spectra of pure PA66 and PA66/Au@Cu_2_O-ZnO composites. The distinctive peaks associated with polyamide can be observed at 3304 cm^−1^ (N–H), 2934 cm^−1^ (–CH_2_–), 2860 cm^−1^ (–CH_2_–), 1629 cm^−1^ (C=O), and 1540 cm^−1^ (N–H), which are in good agreement with the reported literature [32]. Notably, the spectra of the PA66/Au@Cu_2_O-ZnO composite closely resemble those of pure PA66. The absence of new peaks and the IR inactive of the heterojunction indicate the lack of chemical reactivity between Au@Cu_2_O-ZnO and PA66.It is noteworthy that, as the content of the heterostructure increased, the C=O stretching vibration band of PA66 decreased relative to the –CH_2_ band. This is due to the coordination of metal ions with the C=O peak, which reduced the stretching vibration of C=O. This alteration affected the intensity of the characteristic peak at 1629 cm^−1^ [33,34].

Figure 5b shows the decreasing relative viscosity of PA66 along with the increasing Au@Cu_2_O-ZnO heterojunction content. This viscosity reduction can be attributed to the heterojunction disrupting the structural coherence of the polymer chains. The more complex polymerization formed more low-molecular-weight byproducts, cumulatively decreasing the overall viscosity.

**Figure 6:**
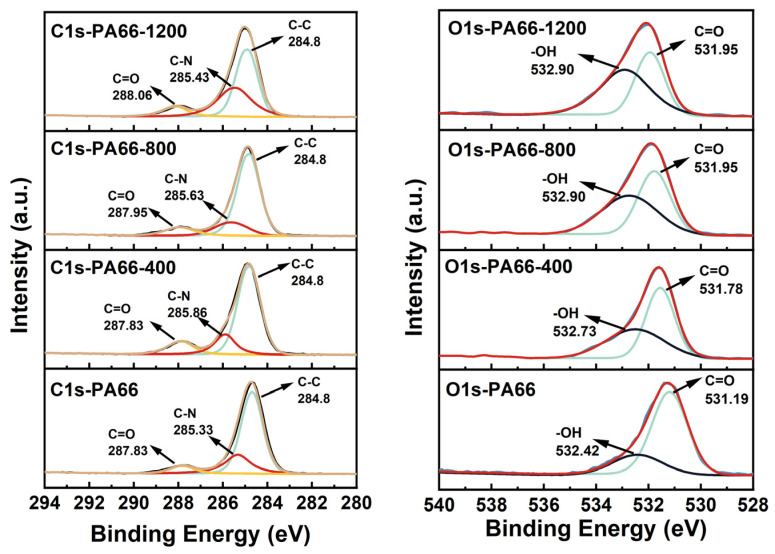
C 1s and O 1s XPS spectra of PA66 and PA66/Au@Cu_2_O-ZnO composites.

XPS tests were also performed to confirm the formation of coordination bonds in the PA66 composites. The C 1s and O 1s XPS spectra of the pure PA66 and PA66/Au@Cu_2_O-ZnO composites are shown in Figure 6. It can be observed that, in heterogeneous structured nanomaterials, there was a strong coordination interaction (metal coordination bonds) between metal ions and oxygen functional groups. As a result, compared to regular PA66, in PA66 formed with the participation of heterogeneous structured nanomaterials, the XPS characteristic peaks of oxygen functional groups shifted towards higher binding energies. In the case of PA66 formed with 400 ppm of heterogeneous structured nanomaterials, the integrated areas of hydroxyl (–OH) and carbonyl (C=O) O1s peaks significantly increased. Moreover, the hydroxyl (–OH) oxygen characteristic peak shifted from 532.42 eV to 532.73 eV, and the carbonyl (C=O) oxygen characteristic peak shifted from 531.19 eV to 531.78 eV [35].

With an increase in the content of nanomaterials in the polymerization process, the corresponding concentration of metal ions in polyester fibers or slices also increased. The effect of metal ions on oxygen functional groups was enhanced, causing further shifts of XPS characteristic peaks of oxygen functional groups in polyester fibers towards higher binding energies. As the content of heterogeneous structured nanomaterials increased from 400 ppm to 1200 ppm, the hydroxyl (–OH) peak shifted from 532.73 eV to 532.9 eV, and the carbonyl (C=O) peak shifted from 531.78 eV to 531.9 eV. Due to the strong coordination interaction between oxygen atoms in oxygen functional groups and metal ions, neighboring carbon atoms were also affected, leading to changes in their XPS characteristic peaks. As the content of heterogeneous structured nanomaterials increased from 0 to 1200 ppm, the integrated area of the carbonyl (C=O) peak also significantly increased, and the carbon characteristic peak of carbonyl (C=O) shifted from 287.83 eV to 288.06 eV [36,37]. The above analysis indicates that heterogeneous structured nanomaterials were involved in an in situ polymerization process rather than simply being doped into PA66.

### 3.3. Crystallization and Melting Behaviors of PA66/Au@Cu_2_O-ZnO Composites

**Figure 7:**
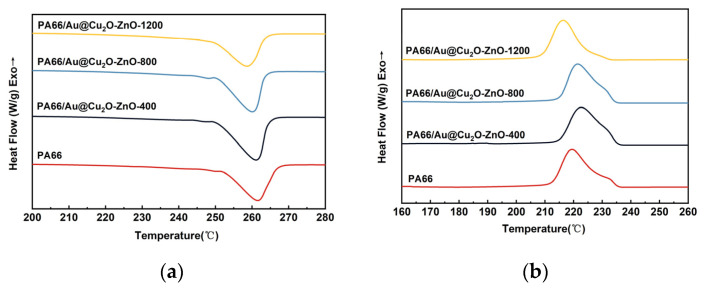
DSC heating curves (**a**) and cooling curves (**b**) of PA66 and PA66/Au@Cu_2_O-ZnO composites.

The DSC curves of pure PA66 and PA66/Au@Cu_2_O-ZnO composites are presented in Figure 7, and the results are summarized in Table 1. With the increasing content of heterojunction antibacterial agents, the melting peak of PA66/Au@Cu_2_O-ZnO composites shifted to lower temperatures and gradually broadened. However, the crystallization peak shifted to higher temperatures initially before decreasing again at a higher heterojunction content. This was due to the introduced heterojunction reducing the structural symmetry and regularity of PA66 molecular chains, hindering the growth of folded macromolecular chains into large crystals and leading to a lowered melting point [32]. The crystallinity of these samples, calculated and shown in Table 1, increased initially with the Au@Cu_2_O-ZnO content before decreasing. This may be because, at low contents, the evenly dispersed Au@Cu_2_O-ZnO nanoparticles can undergo heterogeneous nucleation, inducing crystallization acceleration. However, excessive Au@Cu_2_O-ZnO nanoparticles can impede molecular chain movement.

**Figure 8:**
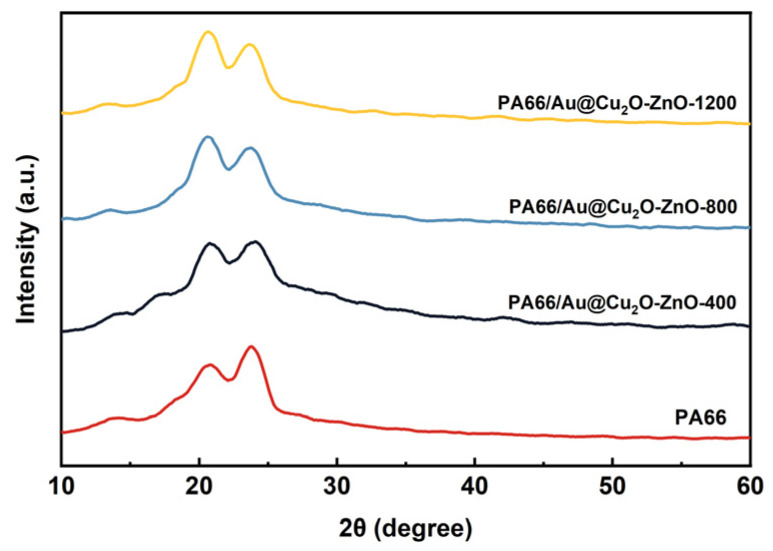
XRD patterns of PA66 and PA66/Au@Cu_2_O-ZnO composites.

In Figure 8, two characteristic diffraction peaks, (100) and (010,110) of α phase crystals, can be observed for pure PA66, PA66/Au@Cu_2_O-ZnO-400, PA66/Au@Cu_2_O-ZnO-800, and PA66/Au@Cu_2_O-ZnO-1200 at 2θ angles of 20.8° and 23.8°, 20.7° and 23.9°, 20.5° and 23.7°, and 20.5° and 23.8°, respectively [38]. With increased heterojunction loading, these peaks shifted to lower angles. The α1 peak’s relative intensity grew, eventually surpassing the α2 peak’s height. This likely resulted from heterojunction complexation with PA66, altering hydrogen bonds between molecular chains and promoting α1 surface growth. However, the α2 plane’s crystal growth remained unaffected due to unchanged van der Waals forces [39,40,41].

### 3.4. Thermal Stability of PA66/Au@Cu_2_O-ZnO Composites

**Figure 9:**
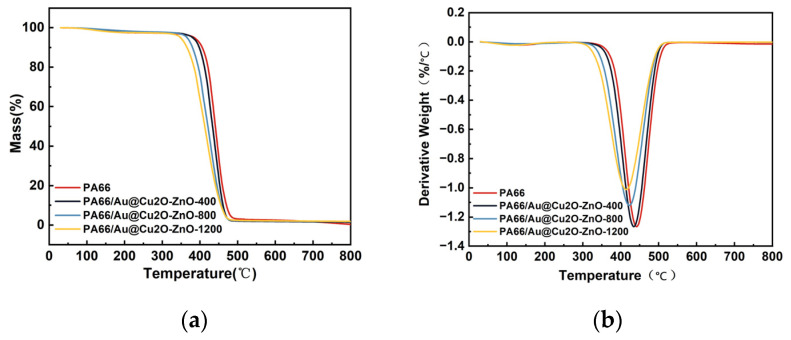
TG and DTG curves of PA66 and PA66/Au@Cu_2_O-ZnO composites under N_2_: (**a**) TG curves; (**b**) DTG curves.

The thermal stability of PA66 and PA66/Au@Cu_2_O-ZnO composites were evaluated using TG and DTG. Figure 9 shows TG and DTG thermograms of PA66 and PA66/Au@Cu_2_O-ZnO composites, and the relevant thermal parameters for PA66 and its nanocomposites are presented in Table 2. T5% was the temperature corresponding to the loss of 5 wt%, that is, the initial decomposition temperature, which also indicates the thermal stability of the material. From Figure 9a, it is evident that PA66/Au@Cu_2_O-ZnO composites had lower T5% than pure PA66. The initial decomposition temperature of PA66/Au@Cu_2_O-ZnO composites was still above 340 °C. The residual carbon ratio of the polymer increased with the increasing heterojunction content. The carbon residue of PA66/Au@Cu_2_O-ZnO composites containing 1200 ppm of heterojunction was 1.88% at 800 °C, and the carbon residue of pure PA66 was 0.37%, suggesting that the PA66/Au@Cu_2_O-ZnO composites had relatively good thermal stability.

The DTG curves (Figure 9b) show that all samples exhibited a single degradation process. The DTG peak (Tmax), representing the temperature of the maximum weight loss rate, was found at 443 °C for pure PA66, corresponding to a maximum loss rate of 1.3 wt%/°C. Further analysis revealed that the PA66/Au@Cu_2_O-ZnO composites had lower maximum weight loss rates than pure PA66, with the composite infused with 1200 ppm heterojunction exhibiting the lowest rate of 1.0 wt%/°C. This evidences that the Au@Cu_2_O-ZnO, as a weak alkaline, can catalyze the degradation of PA66 and decrease the thermal stability of PA66/Au@Cu_2_O-ZnO composites [42,43].

### 3.5. Mechanical Properties of PA66/Au@Cu_2_O-ZnO Composites

**Figure 10:**
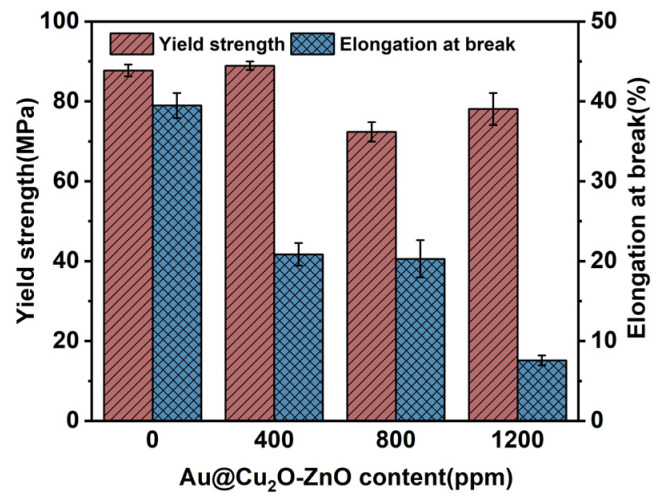
Yield strength and elongation at break of PA66 and PA66/Au@Cu_2_O-ZnO composites.

The mechanical properties of PA66 and the PA66/Au@Cu_2_O-ZnO composites, as shown in Figure 10, reveal that the integration of the Au@Cu_2_O-ZnO heterojunction into PA66 led to a decrease in both yield strength and fracture elongation with the increasing heterojunction content. Specifically, the yield strength and fracture elongation rate declined from 87.76 MPa and 23.54% for pure PA66 to 78.07 MPa and 7.58% for the PA66/Au@Cu_2_O-ZnO composite with 1200 ppm of the heterojunction. This gradual reduction in mechanical properties likely occurred due to the interference of the heterojunction in the polymerization process of PA66, hindering the growth of molecular chains and resulting in a decreased relative molecular mass. Moreover, the heterojunction promoted increased branching in the polymer chains, further diminishing the yield strength and elongation at break. Despite this reduction, the PA66/Au@Cu_2_O-ZnO composite maintained adequate structural integrity, making it a suitable plastic material, especially in applications where antibacterial properties are essential alongside moderate mechanical strength [44].

### 3.6. Evaluation of Antibacterial Performance for PA66/Au@Cu_2_O-ZnO Composites

To evaluate antimicrobial performance, clinically isolated *Escherichia coli* (ATCC 25922, Gram-negative) and *Staphylococcus aureus* (ATCC 6538, Gram-positive) were tested, as they are associated with medical infections [45].

In Table 3, the antibacterial efficacies of both standard PA66 and PA66/Au@Cu2O-ZnO composites against *Escherichia coli* and *Staphylococcus aureus* are compared under varying conditions of darkness and light over a 24-hour period. It is evident from the data that regular PA66 exhibited no significant antibacterial activity against either bacterium. However, when the heterojunction concentration was at a lower level of about 400 ppm, the composites showed a modest degree of antibacterial activity, more so for *Escherichia coli*, even in a dark environment. This activity increased substantially with higher concentrations of the heterojunction, particularly above 800 ppm, where the composites displayed excellent antibacterial properties under light conditions and were notably effective against *Escherichia coli* in the dark. This enhanced performance under light is in line with the known photocatalytic mechanisms of the heterojunction, which rely on electron–hole transitions requiring sufficient energy excitation. These differences in antibacterial activity under varying conditions and heterojunction concentrations are detailed in Figure S5.

## 4. Conclusions

In this work, we successfully synthesized a series of antibacterial polyamide 66 (PA66) composites through an in situ polymerization process. This involved the integration of a ternary heterojunction as the antibacterial agent. X-ray photoelectron spectroscopy (XPS) analyses confirmed the complexation of the heterojunction’s metal ions with the oxygen-containing functional groups within the polymer matrix. Scanning electron microscopy (SEM) and transmission electron microscopy (TEM) examinations revealed that the antibacterial agent was homogeneously dispersed throughout the PA66 matrix. This uniform dispersion was attributed to the formation of metal–ion complexes and hydrogen bonding interactions with the polymer matrix. Furthermore, X-ray diffraction (XRD) studies indicated an increase in the crystallinity of PA66 with higher loading of the heterojunction. This suggests that the heterojunction facilitated the crystalline growth of PA66, likely due to the formation of these complexes. Impressively, the mechanical properties of the composites remained robust despite the addition of the antibacterial agent. Specifically, the yield strength of the PA66/Au@Cu_2_O-ZnO composites containing 1200 ppm of the heterojunction was measured at 77.2 MPa, demonstrating the material’s strength and durability. Antibacterial tests conducted on these composites showed strong inhibition of both *Escherichia coli* and *Staphylococcus aureus*, particularly under light conditions. This highlights the potential of PA66/Au@Cu_2_O-ZnO composites as effective antibacterial agents in illuminated environments. These properties make them suitable for applications such as disinfecting surface coatings, as they can effectively inhibit bacterial growth.

The successful development of these antibacterial PA66 composites not only expands the potential applications of PA66, but also opens avenues for further exploration in the field of antibacterial textiles. This advancement is particularly significant in developing materials for health and safety applications, where controlling bacterial growth is crucial.

## Figures and Tables

**Table 1:** The DSC results of PA66 and PA66/Au@Cu_2_O-ZnO composites.

Sample	Tm, °C	ΔHm, J g^−1^	Tc, °C	Xc, %
PA66	261.62	49.65	219.60	26.13
400 ppm	261.03	60.42	222.65	31.80
800 ppm	260.10	54.19	221.47	28.52
1200 ppm	258.70	43.78	216.41	23.04

**Table 2:** TG and DTG data under N_2_ atmosphere.

Sample	T_5%_, °C	Tmax, °C	Char Residue at 600 °C	Char Residue at 800 °C
PA66	385	441.6	2.55%	0.37%
400 ppm	381.4	435	1.83%	1.46%
800 ppm	365.7	423.3	2.04%	1.77%
1200 ppm	348.3	413.6	2.17%	1.88%

**Table 3:** The antibacterial tests against *Escherichia coli* and *Staphylococcus aureus* with different samples.

Bacterium	Sample	Antibacterial Rate (%)	
		Under Light	No Light
*E. coli*	PA66	No effect	No effect
	PA66/Au@Cu_2_O-ZnO-400	15.73	44.18
	PA66/Au@Cu_2_O-ZnO-800	>99	98.63
	PA66/Au@Cu_2_O-ZnO-1200	>99	>99
*S. aureus*	PA66	No effect	No effect
	PA66/Au@Cu_2_O-ZnO-400	96.36	16.67
	PA66/Au@Cu_2_O-ZnO-800	>99	70
	PA66/Au@Cu_2_O-ZnO-1200	>99	72.78

## Data Availability

The data presented in this study are available upon request from the corresponding author.

## Supplementary Materials

The following supporting information can be downloaded at: https://www.mdpi.com/article/10.3390/polym16010158/s1, Figure S1: Morphology analysis of Au@Cu_2_O: (a) TEM image of Au. (b) SEM image of Au@Cu_2_O. (c) TEM image of Au@Cu_2_O. (d,e) HRTEM and SAED image of Au@Cu_2_O; Figure S2. XRD patterns of Au@Cu_2_O@ZnO; Figure S3. SEM images of fracture sections: (a) PA66/Au@Cu_2_O-ZnO. (b–e) Elemental mapping images of (b) C, (c) O, (d) N, (e) Cu, and (f) Zn; Figure S4. EDS spectra of fracture sections; Table S1. Element content of fracture sections; Figure S5. Antibacterial tests of different samples against *Escherichia coli* and *Staphylococcus aureus*.
