# Supplementary material for: In Situ Polymerization of Antibacterial Modification Polyamide 66 with Au@Cu2O-ZnO Ternary Heterojunction
Source: Polymers (Basel). 2024 Jan 4;16(1):158. doi: 10.3390/polym16010158 (PMC10780995; doi:10.3390/polym16010158)
Supplement: Supplementary file 1 [file polymers-16-00158-s001.zip › polymers-2756603-supplementary.pdf]

Supplementary Materials

In-situ polymerization of antibacterial modification polyamide 66 with Au@Cu_2O-ZnO ternary heterojunction

Xiang Li [1], Mi Zheng [2], Shikun Zhao [3], Zhiwen Cao [3], Kai Pan [3], Xinxing Feng [1], Hua Zhang [1,*] Min Zheng[2,*]and Cheng Wang[2]

1 Institute of System Engineering, Academy of Military Sciences, People's Liberation Army, Beijing 100010, China; 13220298273@163.com (X.L.); feng-xinxing@hotmail.com(F.X.)
2 College of Textile and Clothing Engineering, Soochow University, Suzhou, Jiangsu 215123, China; zhengm666@foxmail.com(Mi Zheng); wangcheng971027@163.com(C.W.)
3 College of Materials Science and Engineering, Beijing University of Chemical Technology, Beijing 100029, China; wsadzsk@live.com (S.Z.); caozhiwen0514@163.com(Z,C.); pankai@mail.buct.edu.cn(K,P.)
* Correspondence: stone1992@126.com (H.Z.); zhengmin@suda.edu.cn (Min Zheng)

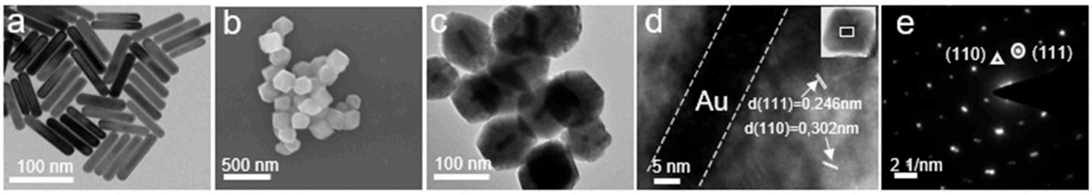

Fig. S1. Morphology analysis of Au@Cu_2O:(a) TEM image of Au. (b)SEM image of Au@Cu_2O. (c) TEM image of Au@Cu_2O. (d,e) HRTEM and SAED image of Au@Cu_2O.

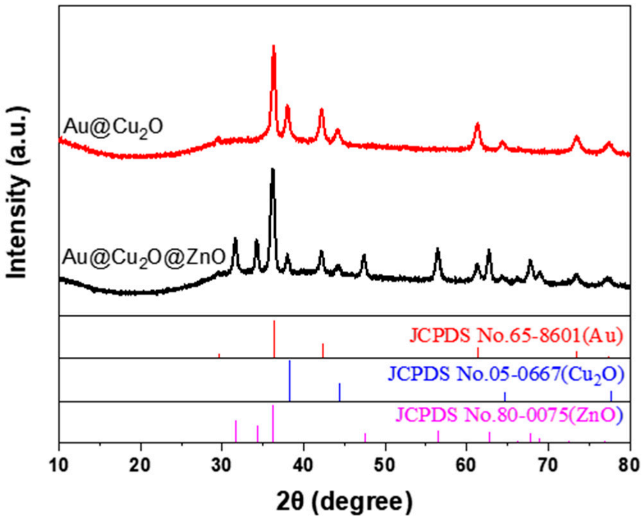

Fig. S2. XRD patterns of Au@Cu_2O@ZnO.

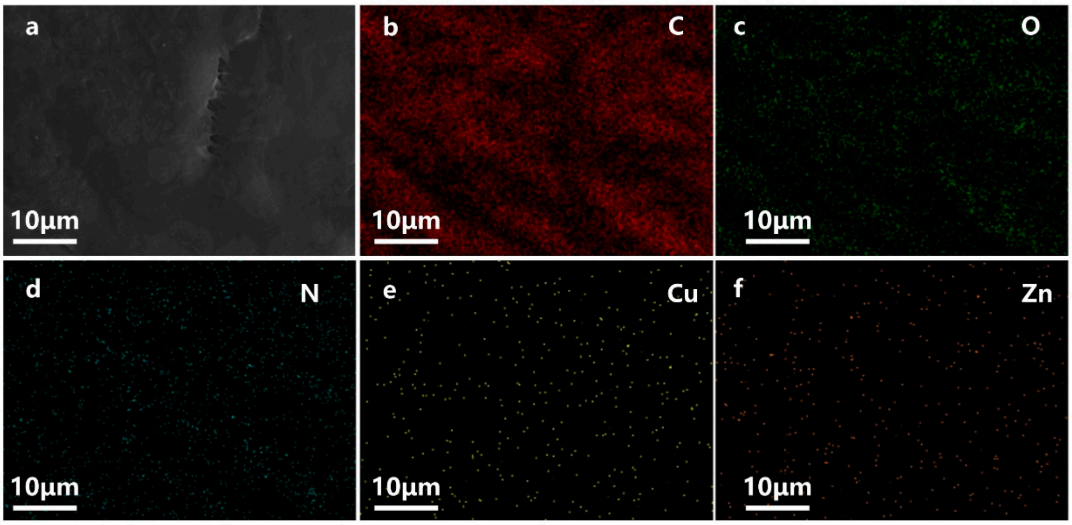

Figure S3. SEM images of fracture sections: (a) PA66/Au@Cu_2O-ZnO; (b–e) Elemental mapping images of (b) C, (c) O, (d) N, (e) Cu, and (f) Zn

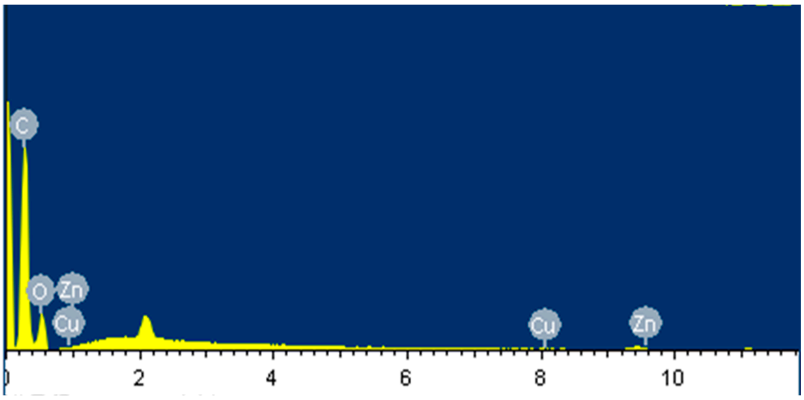

Figure S4. EDS spectra of fracture sections

Table S1 Element content of fracture sections

Element	Weight,%	Atom,%
C	66.35	72.79
O	32.84	27.05
Cu	0.19	0.04
Zn	0.62	0.12
Total	100.00	

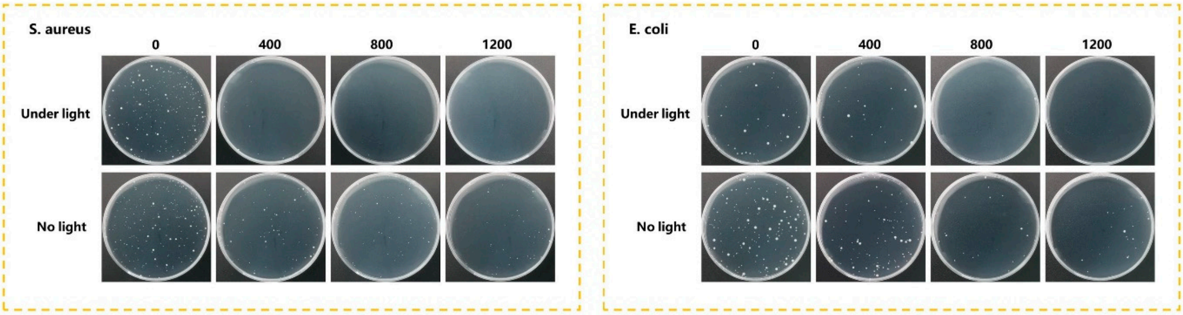

Fig. S5. The antibacterial test against Escherichia coli and Staphylococcus aureus of different samples.
